# Population dynamics and resource availability drive seasonal shifts in the consumptive and competitive impacts of introduced house mice (*Mus musculus*) on an island ecosystem

**DOI:** 10.7717/peerj.13904

**Published:** 2022-09-22

**Authors:** Michael J. Polito, Bret Robinson, Pete Warzybok, Russell W. Bradley

**Affiliations:** 1 Department of Oceanography and Coastal Sciences, Louisiana State University, Baton Rouge, LA, United States of America; 2 Woods Hole Oceanographic Institution, Woods Hole, MA, United States of America; 3 Department of Entomology, San Jose State University, San Jose, CA, United States of America; 4 Point Blue Conservation Science, Petaluma, CA, United States of America; 5 Santa Rosa Island Research Station, California State University Channel Islands, Camarillo, CA, United States of America

**Keywords:** Invasive species, Stable isotope analysis, Seasonality, Island ecology, Diet, Rodents, Seabirds

## Abstract

**Background:**

House mice (*Mus musculus*) are widespread and invasive on many islands where they can have both direct and indirect impacts on native ecological communities. Given their opportunistic, omnivorous nature the consumptive and competitive impacts of house mice on islands have the potential to vary over time in concert with resource availability and mouse population dynamics.

**Methods:**

We examined the ecological niche of invasive house mice on Southeast Farallon Island, California, USA using a combination of mouse trapping, food resource surveys, and stable isotope analysis to better understand their trophic interactions with native flora and fauna. Specifically, we coupled the analysis of seasonal variation in resource availability over a 17-year period (2001–2017), carbon (*δ*^13^C) and nitrogen (*δ*^15^N) stable isotope values of mouse tissue and prey resources in a single year (2013), and isotopic niche and mixing models to quantify seasonal variation in mouse diets and the potential for resource overlap with native species.

**Results:**

We found that plants were the most important resource for house mice during the spring months when vegetation is abundant and mouse populations are low following heavy precipitation and declines in mouse abundance during the winter. While still consumed, plants declined in dietary importance throughout the summer and fall as mouse populations increased, and seabird and arthropod resources became relatively more available and consumed by house mice. Mouse abundance peaks and other resource availability are low on the island in the fall months when the isotopic niches of house mice and salamanders overlap significantly indicating the potential for competition, most likely for arthropod prey.

**Discussion:**

Our results indicate how seasonal shifts in both mouse abundance and resource availability are key factors that mediate the consumptive and competitive impacts of introduced house mice on this island ecosystem. As mice consume and/or compete with a wide range of native taxa, eradication has the potential to provide wide-reaching restoration benefits on Southeast Farallon Island. Post-eradication monitoring focused on plant, terrestrial invertebrate, salamander, and seabird populations will be crucial to confirm these predictions.

## Introduction

House mice (*Mus musculus*) and other rodents are some of the most widespread invasive mammals on earth; amongst vertebrates, the breadth of their global distribution is second only to that of humans (Bronson, 1979; Brooke, Hilton & Martins, 2007). In island ecosystems, house mice have been shown to have direct and indirect ecological impacts on plant, invertebrate, small mammal, and avian communities (Angel, Wanless & Cooper, 2009; Harris, 2009; St Clair, 2011). These impacts can be particularly substantial on islands lacking other invasive rodents such as rats (*Rattus spp.*) and/or where rat eradication efforts have freed mice from the constraints of competition and predation (Broome et al., 2019; Simberloff, 2009). In addition, efforts to eradicate mice from islands are historically less successful than those targeted at *Rattus spp.*, though successful outcomes have become common in recent years (MacKay et al., 2011; MacKay, Russell & Murphy, 2007; USFWS, 2019). Despite this, there has been less research and conservation action devoted to invasive house mice on islands, relative to other introduced mammals (Angel, Wanless & Cooper, 2009; Howald et al., 2007; Wanless et al., 2012; Wanless et al., 2007).

Southeast Farallon Island (SEFI; 37.6989°N, 123.0034°W) is located 48 km west of San Francisco off the coast of central California. This 28 ha island is the largest of the South Farallon Islands, part of the Farallon Islands National Wildlife Refuge, which hosts the largest seabird breeding colony in the contiguous United States (Johns & Warzybok, 2019). SEFI also hosts an introduced house mouse population (USFWS, 2019). Though the exact timing of the introduction of house mice to the South Farallon Islands is unknown, it likely occurred unintentionally during the 1800s or early 1900s (Ainley & Lewis, 1974). While early 20th century data on SEFI mouse abundance are lacking, mice have had a significant presence on the islands from at least the late 20th century to the present (Ainley & Boekelheide, 1990). Closed capture modeling from a mark recapture study on SEFI during near peak fall abundance provided a density estimate of 1,297 ± 224 mice per ha (95% CI: 799–1,792), one of the highest reported mouse densities for any island in the world (Grout & Griffiths, 2013; USFWS, 2019). Commonly, island house mouse densities range from 10 to 50 per ha (MacKay et al., 2011).

Seabirds are particularly sensitive to invasive mammals on islands (Jones et al., 2008). While house mice on islands are known to depredate seabird eggs and chicks and in some cases adults (Bolton et al., 2014; Dilley et al., 2015; Jones et al., 2019), there is little evidence of direct predation by mice on breeding seabirds on SEFI. Despite over 50 years of continuous, intensive study of breeding seabirds, few mouse-depredated eggs or chicks have been detected (Ainley & Boekelheide, 1990; Point Blue Conservation Science, unpublished data, 2022). While predation on eggs by mice can be difficult to detect in many of the crevice-nesting species found on SEFI, these observations suggest the frequency of direct seabird egg or chick predation in this population may be low (Ainley & Boekelheide, 1990). Even so, at a minimum, scavenging of dead seabirds by house mice on SEFI is highly likely based on prior studies of invasive rodents on islands (Angel, Wanless & Cooper, 2009). Moreover, the presence of house mice facilitate migratory burrowing owls (*Athene cunicularia*) to overwinter on SEFI (Chandler et al., 2016; Mills, 2016). When mouse populations seasonally crash, burrowing owls switch from feeding primarily on mice to adult ashy storm-petrels (*Hydrobates homochroa*) which results in significant predation on this species of conservation concern (Nur et al., 2019).

While less studied relative to seabirds, house mice likely also have direct and indirect impacts on the ecological community on SEFI. Jones & Golightly (2006) examined the stomach contents of 57 house mice on SEFI in 2002 and 2003. They found native plants such as the maritime goldfield (*Lasthenia maritima*) constituted 63% of recovered plant material in mouse stomachs (Coulter & Irwin, 2005; Jones & Golightly, 2006). This contrasts with the significantly greater percentage of non-native (63–80%) plant species on SEFI, which is high given the island’s small size and relative isolation (Coulter & Irwin, 2005; Hawk, 2015; Jones & Golightly, 2006). Mice stomachs also contained native invertebrates, including the Farallon camel cricket (*Farallonophilus cavernicolus*) which is endemic to SEFI (Jones & Golightly, 2006; Rentz, 1972). Even so, the interpretation of prey importance and seasonal variation in house mice diets was hampered by the inability to identify and quantify digested prey remains and an inability to sample mice between April to August (Jones & Golightly, 2006). Moreover, no studies have examined the potential impacts of house mice on the endemic Farallon arboreal salamander (*Aneides lugubris farallonensis*). As salamanders feed primarily on insects and other small invertebrates (Bury & Martin, 1973), it is possible that mice and salamanders compete for prey resources as mice have been found to act as resource competitors to native species on other islands (Russell et al., 2020). Given the opportunistic, omnivorous diet of house mice (Berry, 1968; Jones & Golightly, 2006), it is also possible that mice consume salamander eggs when they are laid in the summer and/or small juveniles when they emerge in the fall (Boekelheide, 1975).

Stable isotope analysis represents an alternative approach to traditional dietary analysis which can be used to quantify the ecological niche of native and invasive species and answer ecological questions that were previously intractable (McCue et al., 2020). This approach is based on the principle that animals “are what they eat” with the carbon (*δ*^13^C) and nitrogen (*δ*^15^N) stable isotopes values of consumer tissues reflecting the abundance of these same biomarkers in their food sources (DeNiro & Epstein, 1978; DeNiro & Epstein, 1981). Stable isotope analysis therefore provides insights into species’ “isotopic niche”, which is analogous to the “Hutchinsonian niche” (Hutchinson, 1978), as consumer tissue stable isotope values are directly influenced by what they consume (bionomic) as well as the habitat (scenopoetic) in which they live (Newsome et al., 2007). Furthermore, consumer and prey tissue stable isotope values can be incorporated into dietary mixing models to provide quantitative predictions of consumer diet compositions (Phillips et al., 2014). As consumer tissues integrate dietary information at the time of tissue synthesis it is possible to quantify consumer diets over differing time periods by examining tissues that differ in their rate of metabolic turnover (Hobson & Clark, 1992; Van der Zanden et al., 2015).

The goal of our study was to couple an analysis of seasonal variation in resource availability over a 17-year period with a quantitative assessment of the diets of house mice on SEFI derived from stable isotope analysis to better understand the trophic interactions of mice with native flora and fauna ahead of their proposed eradication by the United Stated Fish and Wildlife Service (USFWS, 2019). Specifically, using isotopic niche and dietary mixing model approaches we quantified seasonal shifts in the diet of house mice in 2013 to determine how the consumptive impacts to bird, arthropod, plant, and intertidal communities vary in concert with mouse abundance and resource availability. Furthermore, we used the isotopic niche approach to quantify the potential for competitive interactions between introduced house mice and endemic arboreal salamanders on SEFI.

## Methods

### Study site

**Figure 1:**
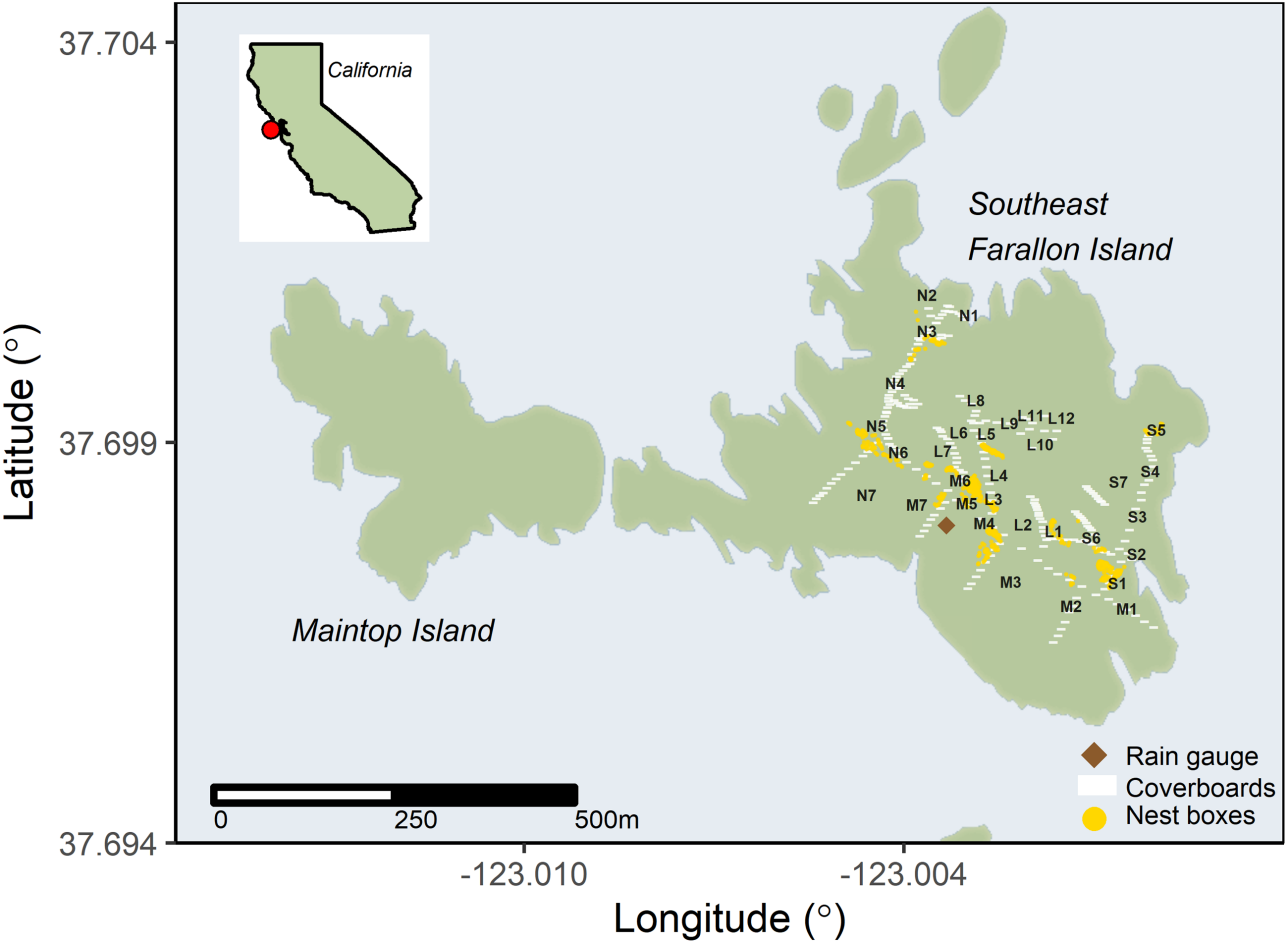
Invasive house mice (*Mus musculus*) trap locations on Southeast Farallon Island. The locations of house mouse traps, the rain gauge used for precipitation measurements, cover boards for arthropod and salamander surveys, and Cassin’s auklet (*Ptychoramphus aleuticus*) nesting boxes on Southeast Farallon Island, CA. Trap locations are noted by their transect letter and trap number. Cassin’s auklets also breed in natural burrows across the island. Other seabird species nest near mouse trapping locations, especially western gulls (*Larus occidentalis*) which are ubiquitous throughout the island.

As described by Chandler et al. (2016), SEFI is the largest of the Farallon Islands, which is one of several islands that compose the Farallon Islands National Wildlife Refuge. Topographically, it is characterized by a 90 m high hill that rises from the center of the island, and a wide, flat marine terrace that extends outward from the base of the hill (Fig. 1). The terrace is widest from the southeastern to the western portions of the island, and seabirds have excavated numerous burrows within its friable soil. The hill is composed of crumbling granite cliffs that contain fissures, crevices, caves, and rocky scree fields at its base. SEFI has a temperate, maritime climate, with relatively wet winters and dry summers (average annual rainfall = 51 cm) (Kim, Nelson & Seo, 2009). The air temperature is warmest in October (average: 16.1  °C) and coldest in January (average: 11.4  °C; Kim, Nelson & Seo, 2009).

Over 25% of California’s breeding marine birds, with nearly 400,000 individuals of 13 species, are found on the South Farallon Islands (Carter et al., 1992; Johns & Warzybok, 2019; Karl et al., 2001). Most areas on SEFI are occupied continually by breeding seabirds between late March and mid-August with cormorants (*Urile penicillatus*, *Urile pelagicus, Nannopterum auritum*) and common murres (*Uria aalge*) inhabiting rocky slopes and cliffs, storm-petrels (*Hydrobates homochroa, Hydrobates leucorhous*), auklets (*Ptychoramphus aleuticus, Cerorhinca monocerata*), pigeon guillemots (*Cepphus columba*), and tufted puffins (*Fratercula cirrhata*) nesting in rock crevices and burrows, black oystercatchers (*Haematopus bachmani*) nesting along the rocky shoreline, and western gulls (*Larus occidentalis*) nesting across the island and most common on the flatter or more gently sloped areas (Ainley & Boekelheide, 1990; Johns & Warzybok, 2019; Karl et al., 2001). Five species of pinniped visit and/or breed on SEFI including the northern elephant seal (*Mirounga angustirostris*), harbor seal (*Phoca vitulina*), Steller sea lion (*Eumetopias jubatus*), California sea lion (*Zalophus californianus*), and the northern fur seal (*Callorhinus ursinus*) (Karl et al., 2001; USFWS, 2009). While hoary bats (*Aeorestes cinereus*), Mexican free-tail bats (*Tadarida brasiliensis*), and Western red bat (*Lasirurs blossevillii*), have been recorded visiting the island, house mice are the only breeding terrestrial mammals present on SEFI (Karl et al., 2001; Tenaza, 1966; Point Blue Conservation Science, unpublished data, 2022). The islands flora includes at least 44 species, 26 of which are non-native (Coulter & Irwin, 2005). Honda et al. (2017) reported 11 orders of terrestrial arthropods representing 60 families, 107 genera and 112 species identified on SEFI. Two endemic taxa are found on SEFI: the Farallon camel cricket (Rentz, 1972) and the Farallon arboreal salamander (Van Denburgh, 1905). Domestic cats (*Felis catus*) and European rabbits (*Oryctolagus cuniculus*) were intentionally introduced to SEFI in the late 1800s and successfully removed from the island in the early 1970s (Ainley & Lewis, 1974).

### Ethics statement

Sampling was approved by the United States Fish and Wildlife Service under a cooperative agreement with Point Blue Conservation Science (no. 81640AJ008) and the California Department of Fish and Game scientific permit (no. SC–008556). All vertebrate sampling protocols were approved by and adhered to statutes of the Institutional Animal Care and Use Committee of the Woods Hole Oceanographic Institution (no. 4855-001).

### Mouse abundance and resource availability

We compiled data over a 17-year period (December 2000 to January 2018; hereafter 2001–2017) to quantify seasonal trends in mouse abundance and resource availability on SEFI. This included an index of mouse abundance based on monthly trapping, precipitation data as a proxy for vegetation phenology, and measures of arthropod, seabird, and salamander abundances. For all metrics, monthly values were compiled and averaged to obtain seasonal values for the spring (March, April, May), summer (June, July, August), fall (September, October, November), and winter (December, January, February) for each season and year that data were available (Table S1).

Our index of mice abundance was based on monthly trapping success on 4, ∼300 m transect lines (L, M, N, and S; Fig. 1) spread across available habitats at SEFI (Irwin, 2004; Chandler et al., 2016; Nur et al., 2019). Trapping was conducted for three consecutive nights each month between March 2001 and March 2004, and again from December 2010 to March 2012, and finally September 2016 through January 2018. All sampling periods used the same transects, each with seven traps per transect. For the 2010–2012 and 2016–2018 effort, five additional traps were added to transect L; these incorporated more of the vertical aspect of the island topography (Fig. 1). Trapping efforts used D-Con^®^ Ultra Set^®^ covered snap traps baited with peanut butter and oats. Trapping success was determined as the proportion of trap-nights set per monthly session (either 84 (2001–2004) or 99 (2010–2012 and 2016–2018)) in which house mice were captured.

Precipitation data (cm of rain) was collected on SEFI using a standard US National Weather Service rain gauge (∼1 m elevation) at the same location at noon Pacific Standard Time every day throughout 2001–2017 (Fig. 1). Precipitation data was used as a proxy for both terrestrial environmental conditions and as a leading proxy for vegetation phenology as the majority of vegetation on SEFI senesces or dies during the summer and recovers in the late winter and spring when seasonal rainfall begins (Coulter & Irwin, 2005).

Arthropod density (indiv./m^2^) on SEFI was evaluated during four collecting trips during 2014. Each trip lasted roughly 12 continuous days and fell within a season (winter, spring, summer, fall). Arthropod densities were enumerated by systematically surveying standardized (0.3 m^2^), untreated wooden cover boards placed throughout the island in a previous study to create salamander refuges (Lee et al., 2012). Complete methods of arthropod collection, species identifications, and density estimates are detailed in Honda et al. (2017).

We used daily year-round assessments of adult seabird carcasses found during routine island operations as proxy of seabird resource availability to house mice on SEFI between 2001 and 2017. As operations and research protocols have remained standard throughout the time series, effort is relatively consistent throughout the period. Adult birds had their primary tips clipped to prevent double counting. For the purpose of this analysis, we used carcass count numbers from one common burrow nesting species whose breeding habitat overlapped mouse sampling areas, the Cassin’s auklet (*Ptychoramphus aleuticus*), as a leading indicator of overall seabird resource abundance (Fig. 1). Cassin’s auklets on SEFI initiate breeding in early to middle of April on average (Ainley & Boekelheide, 1990). Other seabirds also breed in the areas sampled for mice on SEFI, especially western gulls which are ubiquitous and breed throughout the island (Ainley & Boekelheide, 1990). These other seabirds may also act as food resources to house mice, though they initiate breeding slightly later than Cassin’s auklets on average, in early to middle of May (Ainley & Boekelheide, 1990).

We quantified seasonal variability in the abundance (indiv./month) of Farallon arboreal salamanders on SEFI using data from a long-term monitoring program of this species (Lee et al., 2012). A proxy for salamander abundance was estimated by systematically surveying standardized (0.3 m^2^), wooden cover boards placed throughout the island between 2008 and 2017 (Fig. 1). One hundred cover boards along a path from the north to the south side of the island were consistently monitored in all years and additional 148 cover boards spread around the island were also monitored from 2013 to 2017 (Fig. 1). Complete methods for salamander surveys are detailed in Lee et al. (2012).

Prior to statistical analyses average monthly values were used to calculated seasonal averages for salamander abundance, mouse trapping success, and arthropod density. Total monthly values were compiled to calculate seasonal averages for rainfall and bird carcasses. We then used the Kruskal-Wallis Test with non-parametric post-hoc comparisons to test for differences among seasons in mouse trapping success and the availability of food resources.

### Tissue sampling

To complement our analysis of resource availability between 2001 and 2017, we collected tissue samples form mice and potential prey items in 2013 to quantify seasonal shifts in resource use by house mice and the potential for resource overlap with arboreal salamanders. Specifically, we sampled muscle and liver tissues from house mice (*n* = 63 individuals) during three different seasons in the spring (March), summer (August), and fall (October) in 2013 that reflect the natural oscillation of mouse abundance on SEFI (Irwin, 2006). Mouse liver tissue stable isotope values reflects short term diet on the scale of days to a week with an average half-life of 3.5 to 5.6 days, while muscle tissues stable isotope values reflect diets on the scale of weeks to months with an average half-life of 29.6 to 30.1 days (DeMots et al., 2010). Mice were not collected during the winter months due to logistical constraints. We also collected samples of possible food resources for mice based on those that were abundant on SEFI, have been identified in a prior analysis of mouse stomach contents (Jones & Golightly, 2006), and/or represented likely isotopically unique prey groups (Phillips et al., 2014). Specifically, during the spring (April) and fall (October) of 2013 we collected representative samples of prey items, vegetative tissue from common native (*Lasthenia maritima* and *Spergularia sp*) and non-native (*Malva spp.* and *Plantago coronopus*) plants and four different taxa of whole-bodied terrestrial arthropods (*Coleoptera* larvae, *Farallonophilus cavernicolus*, *Oniscidea* sp., *Araneae* spp.; Table S2). Five to six plant and arthropod samples were collected per taxa in both seasons. As prior studies suggest that invasive rodents can consume resources from the intertidal zone (Navarrete & Castilla, 1993; Newton et al., 2016), we sampled the muscle tissues from an intertidal snail (*Nucella emarginata*) during the spring (April; *n* = 6) and fall (October; *n* = 5) of 2013. We also collected seabird tissues during the summer (August; *n* = 16) once these resources became more readily available on the island (Table S2). This included Cassin’s auklet muscle, Cassin’s auklet and western gull egg membrane, and western gull guano which reflect seabird tissues that are highly abundant on SEFI and/or have been previously recovered from mouse stomachs (Jones & Golightly, 2006). Finally, we sampled tail clips from arboreal salamanders found under cover boards in the spring (April; *n* = 16) and fall (October; *n* = 16) when they are most active on the island. This allowed us to assess the likelihood of house mice consuming arboreal salamanders on SEFI. All samples were stored frozen (−20 °C) prior to processing for stable isotope analysis.

### Stable isotope analysis

All samples were freeze-dried and then homogenized using a mortar and pestle. Dried homogenized muscle, liver and tail clip samples underwent lipid extraction using 2:1 chloroform:methanol. Samples were placed in a glass vial with a solvent volume 10 times greater than sample volume and sonicated in a water bath for 15 min and then decanted. This procedure was repeated for a total of three cycles and the samples were rinsed in DI water and oven dried at 60 °C for 24 h to remove any remaining solvent. We flash-combusted (PDZ Europa ANCA-GSL elemental analyzer) approximately 1.0 mg of each animal tissue sample and 3.0 mg of each plant tissue sample loaded into tin cups to analyze for carbon and nitrogen elemental composition (C:N ratio) and carbon and nitrogen isotopes (*δ*^13^C and *δ*^15^N) through interfaced PDZ Europa 20-20 continuous-flow stable isotope ratio mass spectrometers (CFIRMS). Raw *δ* values were normalized using glutamic acid (G-17; G-9/USGS-41), bovine liver (G-13), peach leaves (G-7) and nylon 5 (G-18) as standard reference materials. Sample precision based on repeated standard reference materials was 0.1‰for both *δ*^13^C and *δ*^15^N. Stable isotope ratios are expressed in *δ* notation in per mil units (‰), according to the following equation:

\begin{eqnarray*}\delta X=[({R}_{\mathrm{sample}}/{R}_{\mathrm{standard}})-1]\cdot 1000. \end{eqnarray*}

where X is ^13^C or ^15^N and R is the corresponding ratio ^13^C /^12^C or ^15^N /^14^N. The R_standard_ values are based on Vienna PeeDee Belemnite (VPDB) for *δ*^13^C and atmospheric N_2_ for *δ*^15^N.

### Isotopic niche analysis

We assessed variation in isotopic niche (Newsome et al., 2007) position, width, and overlap across seasons (*i.e.,* spring, summer, and fall) and tissue (muscle *vs.* liver) in house mice and between species (*i.e.,* house mice and arboreal salamander) within each season using both multivariate and univariate techniques. We compared isotopic niche positions by computing the Euclidean distance (ED) between group centroids (*δ*^13^C and *δ*^15^N bivariate means) following the methods of Turner, Collyer & Krabbenhoft (2010). Isotopic niche positions were considered to be different if the ED between species examined was significantly greater than zero after comparison to null distributions generated by a residual permutation procedure. If niche positions differed, we then examined the results of univariate general linear models (GLM) and Tukey-Kramer multiple comparison tests to determine which axis (*δ*^13^C and/or *δ*^15^N) contributed to niche differences across seasons or between species (Hammerschlag-Peyer et al., 2011). To examine individual consistency in the isotopic niche of house mice we tested for relationships between individual’s liver (*i.e.,* shorter-term dietary signal) and muscle (*i.e.,* longer-term diet signal) stable isotope values using Pearson correlations.

In addition, we explored variation in niche area and overlaps using standard ellipse areas which can be interpreted as a measure of the core isotopic niche of a population (Jackson et al., 2011). We calculated the Bayesian estimation of standard ellipse area (SEA_b_) for each group to compare two-dimensional niche areas of house mice and arboreal salamanders among species and seasons. We then used the resulting Bayesian posterior probability distributions of SEA_b_ estimates in pairwise-tests to identify significant differences in SEA_b_ at the *p* < 0.05 level. Specifically, we calculated the probability of whether posterior SEA_b_ values from one group are different than the posterior SEA_b_ value from the comparison group (Jackson et al., 2011). Lastly, we compared isotopic niche overlap between groups calculated as the proportion of standard ellipse area corrected for sample size (Jackson et al., 2011) for each group that overlap with a comparison group’s standard ellipse area (SEA_c_ overlap). Among other things, this isotopic niche overlap approach provided us with a quantitative estimate of the potential for competitive overlap between mice and salamanders.

### Dietary mixing model analyses

We used univariate (*δ*^13^C or *δ*^15^N) GLM and Tukey-Kramer multiple comparison tests to examine isotopic differences across major prey resources (seabirds, intertidal snails, plants, and arthropods) collected in each season. As not all prey resources (*i.e.,* seabirds) were collected in every season we grouped prey resources by taxa and season into a single factor when conducting GLM analyses. We then used the SIMMR Bayesian mixing model (Parnell & Inger, 2016; Parnell et al., 2010) in the R environment (Ver. 3.6.2) to quantify the relative use of these four major prey resources by house mice in each season. This model estimates the probability distributions of multiple source contributions to a mixture while accounting for the observed variability in source and mixture isotopic signatures, elemental concentration, and dietary isotopic fractionation. We focused our mixing model analyses on liver tissues given the strong correlation in isotopic values found between mouse tissues (see Results below) and the more constrained and shorter isotopic turnover time in liver (DeMots et al., 2010; MacAvoy, Macko & Arneson, 2005). Separate models were run in each season (spring, summer, summer). As the discriminatory power of Bayesian mixing models can decline markedly above six or seven prey sources (Phillips et al., 2014), we *a priori* defined five statistically, ecologically, and taxonomically relevant prey resource groups for incorporation into our mixing model analysis. As little to no differences in prey resource stable isotopes values were observed across seasons (see Results below), we used the same prey resource *δ*^13^C and *δ*^15^N values averaged across all seasons in each model analyses. As there is no existing evidence to support or refute the possibility that house mice may also consume arboreal salamanders on SEFI we also explored a separate subset of mixing models that include arboreal salamanders as a prey resource for house mice. We used diet to consumer isotopic discrimination factors for liver tissue (*δ*^15^N: +4.3 ± 0.2; *δ*^13^C: +0.7 ± 0.3) derived from captive studies of house mice on a controlled diet of wheat and corn (Arneson & MacAvoy, 2005). We incorporated elemental concentration dependence (Phillips & Koch, 2002) into the model, and ran 1 million iterations, thinned by 15, with an initial discard of the first 40,000 resulting in 64,000 posterior draws. Following Bicknell et al. (2020), model convergence and fit were checked using Gelman -Rubi diagnostic values (*i.e.,* Gelman–Rubin statistics <1.1) and by plotting the posterior predictive distributions (Gelman et al., 2004).

## Results

### Seasonal trends in mouse abundance and resource availability

**Figure 2:**
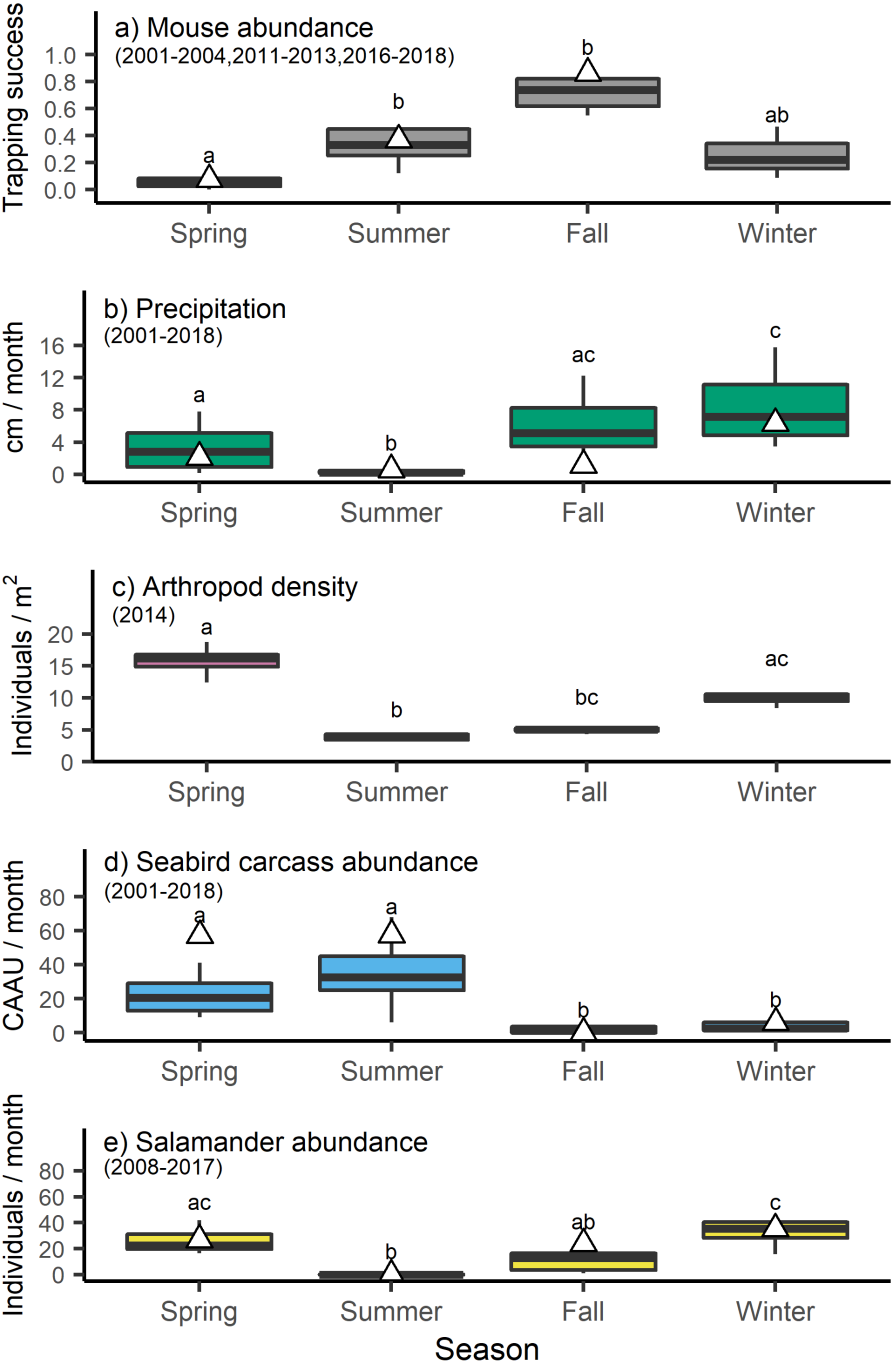
Seasonal shifts in the abundance of invasive house mice (*Mus musculus*), precipitation, and major prey resources. The relative abundance of (A) invasive house mice (*Mus musculus*), (B) monthly precipitation amount, (C) seasonal arthropod densities, (D) monthly seabird carcass abundance (CAAU: Cassin’s auklet (*Ptychoramphus aleuticus*), and (E) monthly arboreal salamanders (*Aneides lugubris farallonensis*) abundance on Southeast Farallon Island, CA during the spring, summer, fall and winter seasons for the years listed. Triangles represent the seasonal mean values in 2013 when tissues were collected for stable isotope analyses. Grey bar and whiskers represent seasonal mean (±SD) values across all of the years listed for each dataset. For each dataset, seasons that share a superscript are not significantly different at the *p* < 0.05 level.

Mouse trapping success varied by season (H_3_ =19.1058, *p* < 0.001) with lower trapping success in the spring relative to the late summer and fall (Fig. 2). These long-term trends broadly concurred with the trapping success observed in 2013, when mouse tissue and food resource samples were collected for stable isotope analyses (Table S1, Fig. 2). Precipitation also differed across seasons (H_3_ =43.8296, *p* < 0.001) being lowest in the summer, intermediate in the spring and fall, and highest in the winter (Fig. 2). Arthropod density varied significantly across seasons (H_3_ =20.2933, *p* < 0.001) with lower values in the summer and higher values in the spring and winter (Fig. 2). Seabird carcass abundance also varied significantly across seasons (H_3_ =47.3676, *p* < 0.001) with higher values in spring and summer relative to fall and winter (Fig. 2). However, seabird carcasses abundance in the spring and summer of 2013 was higher than the long time average. Finally, salamander abundances differed across seasons (H_3_ =30.3015, *p* < 0.001) with higher average counts in spring and winter relative to summer (Fig. 2).

### Isotopic niche of house mice and arboreal salamanders

The isotopic niche position of house mice differed significantly in all pairwise comparisons across seasons for both liver (ED = 4.01−5.37‰, *p* < 0.001) and muscle (ED = 2.00−5.02‰, *p* < 0.030) tissues. House mice stable isotope values differed among seasons (*δ*^13^C: *F*_2,126_ = 66.61, *p* < 0.001; *δ*^15^N: *F*_2,126_ = 26.60, *p* < 0.001) but not by tissue type (*δ*^13^C: *F*_1,126_ = 66.61, *p* = 0.878; *δ*^15^N: *F*_1,126_ = 0.92, *p* = 0.339) or the interactions between these two factors ( *δ*^13^C: *F*_1,126_ = 2.32, *p* = 0.681; *δ*^15^N: *F*_1,126_ = 1.22, *p* = 0.299). Seasonal differences in the isotopic niche position of house mice were due to lower tissue *δ*^13^C values in the spring relative to the summer and fall as well as lower tissue *δ*^15^N values in the summer relative to the spring (liver and muscle) and fall (liver only; Table 1). Muscle and liver tissue stable isotope values were positively correlated with one another for both *δ*^13^C (*r* = 0.908, *p* < 0.001) and *δ*^15^N (*r* = 0.946, *p* < 0.001) values.

**Table 1:** The stable carbon and nitrogen stable isotope values of invasive house mice (*Mus musculus*) and endemic arboreal salamanders (*Aneides lugubris farallonensis*). Mouse and salamander tissue *δ*^13^C and *δ*^15^N values and C/N ratios were measured from individuals sampled during the spring, summer and fall sampling periods on Southeast Farallon Island, CA in 2013. Groups that share a superscript are not significantly different at the *p* < 0.05 level.

Taxa, Tissue	Season	*n*	C/N	*δ*^13^C (‰)	*δ*^15^N (‰)
House mice, liver	Spring	23	3.7 ± 0.1	−24.5 ± 0.6^a^	27.0 ± 1.8^a^
	Summer	20	3.5 ± 0.1	−22.0 ± 1.3^b^	22.2 ± 4.2^b^
	Fall	20	3.5 ± 0.1	−21.2 ± 1.6^bc^	26.1 ± 2.3^a^
House mice, muscle	Spring	23	3.4 ± 0.1	−24.4 ± 1.1^a^	26.8 ± 1.5^a^
	Summer	20	3.4 ± 0.2	−21.8 ± 1.6^b^	22.5 ± 4.6^b^
	Fall	20	3.4 ± 0.1	−21.7 ± 1.4^b^	24.5 ± 1.8^ab^
Arboreal salamander, tail clip	Spring	16	3.4 ± 0.2	−21.5 ± 1.0^b^	25.9 ± 1.4^a^
	Fall	16	3.3 ± 0.1	−20.0 ± 1.4^c^	24.5 ± 1.5^ab^

**Figure 3:**
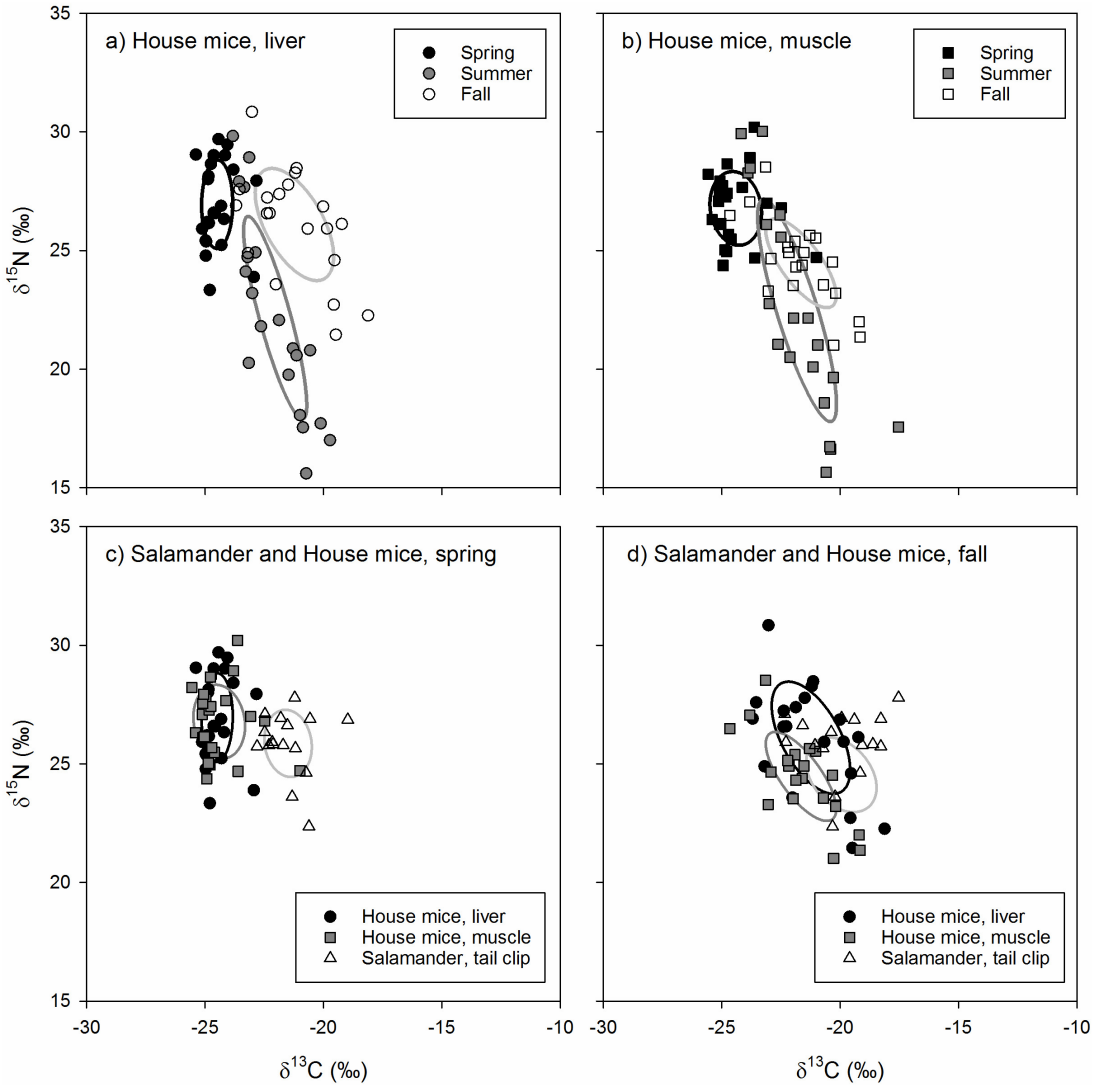
(A–D) The stable isotope values and isotopic niches of invasive house mice (*Mus musculus*) and endemic arboreal salamanders (*Aneides lugubris farallonensis*). The carbon (*δ*^13^C) and nitrogen (*δ*^15^N) stable isotope values and isotopic niche areas of mouse liver and muscle tissues and salamander tail clip tissues collected during the spring, summer and fall sampling periods on Southeast Farallon Island, CA in 2013. Isotopic niche areas are presented a standard ellipse area corrected for sample size (SEA_*c*_; Jackson et al., 2011).

The isotopic niche area of house mice differed significantly across seasons due to smaller SEA_b_ values in spring relative to summer and fall for liver tissues and higher SEA_b_values in summer relative to spring and fall for muscle tissues (Table 2). While the isotopic niche of house mice did not overlap among seasons when examined using liver tissues, niche overlap based on muscle tissue was higher between spring and summer (7.6–18.9%) and spring and fall (11.0–12.4%) relative to fall and summer (0.0−0.1%; Fig. 3).

**Table 2:** Isotopic niche area of invasive house mice (*Mus musculus*) and endemic arboreal salamanders (*Aneides lugubris farallonensis*). Isotopic niches of mice and salamanders sampled during the spring, summer and fall sampling periods on Southeast Farallon Island, CA in 2013 are presented as Bayesian estimation of standard ellipse area (SEA_b_) in square per mil units (‰^2^) and their corresponding 95% credibility intervals. Groups that share a superscript are not significantly different at the *p* < 0.05 level.

Taxa, tissue	Season	*n*	Mean SEA_b_ (‰^2^)	SEA_b_ 95% CI (‰^2^)
House mice, liver	Spring	23	4.05^a^	2.86–5.61
	Summer	20	9.40^bc^	6.46–13.39
	Fall	20	9.54^bc^	6.58–13.51
House mice, muscle	Spring	23	5.20^ab^	3.67–7.25
	Summer	20	12.87^c^	8.90–18.22
	Fall	20	5.88^ab^	4.05–8.42
Arboreal salamander, tail clip	Spring	16	4.37^a^	2.89–6.44
	Fall	16	6.52^abc^	4.29–9.66

The isotopic niche position of house mice and arboreal salamanders differed significantly in all within-season, pairwise comparisons of arboreal salamander tail clips and mouse liver (spring: ED = 3.19‰, *p* < 0.001; fall: ED = 2.02‰, *p* = 0.004) and muscle (spring: ED = 3.05‰, *p* < 0.001; fall: ED = 1.71‰, *p* = 0.011) tissues. This was due to higher *δ*^13^C values in arboreal salamanders’ tail clips relative to mouse liver and muscle tissues in the spring (*F*_2,118_ = 63.61, *p* < 0.001) and muscle tissues only in the fall (*F*_2,118_ = 6.03, *p* = 0.004; Table 1). In contrast, arboreal salamanders tail clip *δ*^15^N values did not differ from house mouse liver and muscle tissues in the spring (*F*_2,118_ = 2.23, *p* = 0.101) or the fall (*F*_2,118_ = 3.40, *p* = 0.057; Table 1).

The isotopic niche area of arboreal salamanders did not differ significantly between seasons, nor did they differ from estimates of house mice niche area calculated from liver or muscle tissues within each season (Table 2). Isotopic niche overlap between spring and fall for arboreal salamanders ranged from 16.0–24.9%. The isotopic niche of arboreal salamanders in the spring did not overlap with those of house mice estimated using either liver or muscle tissues (Fig. 3). In contrast, the isotopic niche of arboreal salamanders in the fall showed significant overlap with the isotopic niche area (SEA_c_) of house mice estimated using liver tissue (16.3%) or muscle tissues (52.3%; Fig. 3).

### Dietary mixing model analyses

**Figure 4:**
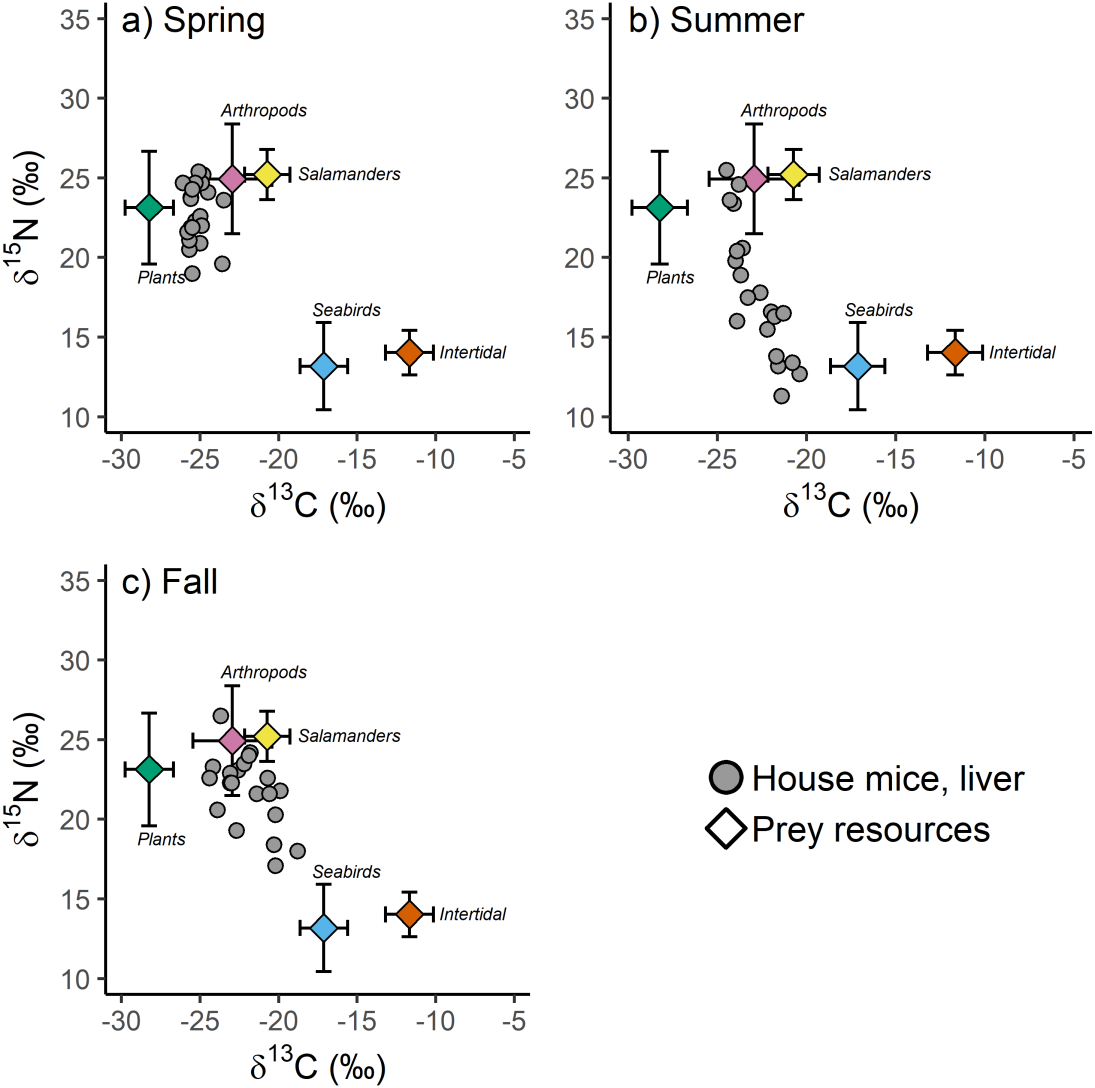
(A–C) The stable isotope values of invasive house mice (*Mus musculus*) and major prey resources. The carbon (*δ*^13^C) and nitrogen (*δ*^15^N) stable isotope values of mouse liver tissues and prey resources in the spring, summer and fall sampling periods on Southeast Farallon Island, CA in 2013. House mice stable isotope values been adjusted by subtracting dietary isotopic discrimination factors for liver (*δ*^15^N: 4.3 ± 0.2; *δ*^13^C: 0.7 ± 0.3) tissues (Arneson & MacAvoy, 2005).

Differences in prey resource stable isotope values between major taxonomic groupings were more common than differences within major taxonomic groupings collected in different seasons for both *δ*^13^C (*F*_4,152_ = 132.60, *p* < 0.001) and *δ*^15^N (*F*_4,152_ = 38.25, *p* < 0.001) values. For example, plant and intertidal resource *δ*^13^C and *δ*^15^N values, and arthropod and arboreal salamander *δ*^15^N values did not differ between seasons (Table S2). Arthropod and arboreal salamander *δ*^13^C values differed slightly between seasons, though these differences were small (1.5−1.9‰) relative to the differences observed between major taxonomic groupings (2.2–16.5‰; Table 1, Table S2). Because of these findings, and because not all prey resources were collected in each season due to logistical and financial constraints even though they were available to mice, prey resource *δ*^13^C and *δ*^15^N values were averaged across all seasons by major taxonomic groupings for subsequent analyses (Table S3). These averaged prey resource *δ*^13^C ( *F*_4,152_ = 237.27, *p* < 0.001) and *δ*^15^N (*F*_4,152_ = 75.35, *p* < 0.001) values differed significantly among major taxonomic groups thus providing isotopically unique endmembers for incorporation into the isotopic mixing model (Fig. 4).

When examined without arboreal salamanders as a possible prey resource, stable isotope mixing model analysis indicate seasonal shifts in the diet composition of house mice on SEFI. While there is overlap in 95% credibility intervals between some groups, plants dominated the diet in the spring, followed by a smaller proportion of arthropods, and very little intertidal and seabird resources (Table 3). The importance of plants decreased in the summer, while the relative importance of seabirds during this time increased to the highest observed across all seasons (Table 3). Fall diets were characterized by a relatively increased importance of arthropod and to a lesser extent intertidal resources, a continued decline in the importance of plants, and a decrease in the importance of seabirds. When prey resources were aggregated *a posteriori*, there were clear differences in the relative use of marine (*i.e.,* seabird and intertidal) *vs.* terrestrial (*i.e.,* plant and arthropod) resources by mice across seasons. While terrestrial resources were more important to mice in all three seasons, the relative importance of marine resources were higher in the summer and fall, relative to the spring (Table 3).

**Table 3:** The predicted diet composition of invasive house mice (Mus musculus) from stable isotope-based mixing models. Diet compositions are reported for the spring, summer and fall sampling periods on Southeast Farallon Island using models both with and without endemic arboreal salamanders (Aneides lugubris farallonensis) as possible prey resources. Diet proportions for each prey resource individually and grouped into marine (seabirds and intertidal) and terrestrial (insects, plants, and salamanders) prey resources, are presented as mean values and 95% creditability intervals in parentheses.

Model, prey sources	Diet proportion (%)		
	Spring	Summer	Fall
Without Salamanders			
Seabirds	3.5 (0.0–7.9)	26.1 (7.9–41.3)	7.2 (0.0–17.6)
Intertidal	8.5 (0.8–15.0)	10.7 (0.0–27.3)	21.4 (6.1–33.9)
Plants	63.0 (54.2–71.5)	41.7 (27.7–58.0)	25.7 (8.7–41.0)
Insects	25.0 (14.3–36.2)	21.5 (1.5–38.4)	45.7 (27.3–66.4)
Total marine	12.0 (8.0–15.8)	36.8 (30.3–43.2)	28.5 (22.2–34.1)
Total terrestrial	88.0 (84.2–92.0)	63.2 (56.8–69.7)	71.5 (65.9–77.8)
With Salamanders			
Seabirds	4.5 (0.4–8.1)	26.2 (11.4–39.6)	10.0 (0.2–19.4)
Intertidal	4.6 (0.0–10.8)	8.2 (0.0–21.6)	14.0 (0.1–27.4)
Plants	60.0 (52.5–67.7)	40.7 (27.3–54.3)	23.2 (6.5–38.8)
Insects	12.1 (0.1–24.1)	14.9 (0.0–32.3)	31.2 (4.2–56.0)
Salamanders	18.9 (4.8–32.6)	10.1 (0.0–24.4)	21.6 (0.3–41.0)
Total marine	9.1 (5.8–12.9)	34.3 (26.8–41.2)	24.1 (17.2–31.0)
Total terrestrial	90.9 (87.1–94.2)	65.7 (58.8–73.2)	75.9 (69.0–82.8)

These same seasonal trends are also apparent when including salamanders as a possible prey resource. Plants are most important in the spring and decline in contribution to the diet throughout the summer and fall (Table 3). Seabird resources are consumed relatively more in the summer and arthropod resources are relatively more important in the fall. Arboreal salamanders are predicted to contribute between 10.1 and 21.6% of house mice diet (Table 3). Mixing models that include arboreal salamanders as a possible prey resource tend to predict lower arthropod contribution to house mice diets than those that do not include salamanders likely due to the fact that these two groups have the most similar stable isotope values of the possible prey resources examined in our study (Table 3, Table S3). When prey resources were aggregated *a posteriori* into marine *vs.* terrestrial resources, the results of models that included salamanders did not differ from those that did not (Table 3).

## Discussion

Our study provides insights into the diet of invasive house mice on Southeast Farallon Island (SEFI). Specifically, the use of stable carbon and nitrogen isotope analysis combined with isotopic niche and dietary mixing model approaches allowed us to quantitatively assess the diets and foraging niches of house mice on Southeast Farallon Island to better understand their interactions with native flora and fauna. We found that plants are the most important resource for house mice in the spring when plants are most abundant and house mouse populations are low (Fig. 2, Table 3). However, as mouse populations increased throughout the summer and fall, seabird and arthropod resources become relatively more available and important to house mice while plant resource use declined (Fig. 2, Table 3). In addition, when the mouse population is high on SEFI and possibly other resources are less abundant, the isotopic niches of house mice and salamanders overlap significantly indicating the potential for competition, most likely for arthropod prey (Figs. 2 and 3). These results indicate how seasonal shifts in both the mouse population and resource availability are key factors that drive the consumptive and competitive impacts of introduced house mice on this island ecosystem.

Our results broadly agree with dietary studies of invasive mice on other island ecosystems. On Antipodes Island, the predatory and competitive impacts of house mice vary with seasons, tracking resource availability from abundant invertebrates and land birds over summer to terrestrial vegetation and seabirds in winter (Russell et al., 2020). Similarly, Bicknell et al. (2020) found that the diets of introduced St Kilda field mouse (*Apodemus sylvaticus hirtensis*) on Hirta varied among sub-populations that had differing spatial access to seabird resources, and between the seabird breeding and non-breeding seasons. Smith, Avenant & Chown (2002) observed both habitat and season-specific variation in the diet of invasive house mice on Marion Island, with plant material most important in summer and invertebrates most important in winter and spring. Furthermore, climate change is predicted to exacerbate the direct and indirect impacts of house mice on Marion Island *via* shifts in mouse abundance, primary productivity, decomposition, and nutrient cycling (Smith, 2002). The impacts of house mice on SEFI are also likely to be affected by climate change, which is predicted to cause increased temperatures, shifts in rainfall patterns, changes in the phenology and composition of island vegetation, and changes to the invertebrate communities (USFWS, 2019). These results and others highlight the highly plastic, omnivorous dietary niche of mice inhabiting island ecosystems and their ability to quickly respond to seasonal and spatial resource pulses (Drever et al., 2000; Le Roux et al., 2002; Quillfeldt et al., 2008; Shiels et al., 2013).

While our study used stable isotope analysis, a prior study of house mouse diet on SEFI relied solely on stomach content analyses (Jones & Golightly, 2006). They found that plants and arthropods were found in mouse stomachs throughout the year, but that eggshell and feather fragments were only recovered in the summer months (Jones & Golightly, 2006). However, stomach contents reflect only a “snapshot” of an individual’s recent diet. Therefore, rodent stomach contents can often be highly variable, biased towards prey that does not readily digest, and may underestimate the amount of soft-bodied prey (Hansson, 1970; Jordan, 2005; Monadjem, 1997). In addition, prey recovered from the stomach contents are often unable to be identified (Jordan, 2005). Because the stable isotope values of mouse tissues integrate dietary information over days to months, they avoid many of the digestive and temporal biases inherent to stomach content analyses (DeMots et al., 2010; MacAvoy, Macko & Arneson, 2005). Even so, the stable isotope-based analyses performed in our study broadly agrees with the seasonal dietary trends in frequency of occurrence observed by Jones & Golightly (2006), while in some cases also providing more constrained estimates of the relative dietary proportion of each prey source.

The mouse tissues analyzed in our study were collected from March 21 to March 29 (*i.e.,* spring), August 13 to August 15 (*i.e.,* summer), and October 26 (*i.e.,* fall). Given an average half-life of 3.5 to 5.6 days for mouse liver tissue (DeMots et al., 2010), the stable isotope values of liver tissue correspond to mouse diets during periods well within the examined three-month window of seasonal resource availability. Muscle tissues have an average half-life of 29.6 to 30.1 days (DeMots et al., 2010). This implies that the stable isotope values of muscle tissue collected during the summer and fall are also primarily reflective of mouse diets during these same seasons as they were collected in the middle and last months of these seasons, respectively. However, muscle tissue collected during the spring may be reflective of some combination of mouse diets during the first month of the spring and the last month of the winter, as they were at the end of the first month of the spring season. The broad similarity in mouse liver and muscle stable isotope values collected in the spring suggests that the late winter diets were similar to the early spring diets. Moreover, the positive correlation between mouse liver and muscle stable isotope values found in our study suggests a degree of individual consistency in mouse diets over time (Bearhop et al., 2004; Herman et al., 2017).

Stable isotope analysis, like stomach content analysis, cannot discern whether the seabird resources consumed by mice were the result of predation or scavenging. During the summer seabird breeding season on SEFI there is ample opportunity for invasive mice to scavenge chick and adult carcasses, lost or deserted eggs, seabird regurgitate, and/or guano due to the high abundance of these resources on the island. Studies on other islands using camera traps, behavioral observations, and other methods have found clear evidence of mice actively predating seabird egg and live chicks (Bicknell et al., 2020; Bicknell, Reid & Votier, 2009; Wanless et al., 2012; Wanless et al., 2007). The little observational data that exist on SEFI suggest the potential for a low degree of direct predation on seabird eggs, chicks, or adults (Ainley & Boekelheide, 1990). However, with the information currently available it is not possible to fully assess the relative occurrence of seabird predation *vs.* scavenging by house mice on SEFI.

Our stable isotope-based mixing model estimates of mouse diets on SEFI have additional assumptions and limitations. For example, we parametrized the mixing model using isotopic discrimination factors for liver tissue derived from captive studies of house mice on a controlled diet (Arneson & MacAvoy, 2005). If the isotopic differences between diets and mouse livers on SEFI varied from those measured in this controlled study, they could bias the resulting estimations of the proportional contribution of each diet component (Bond & Diamond, 2011). However, given that these discrimination factors are tissue and species-specific, and that mouse tissue isotopic values were well within the range of the dietary resources examined, there is sufficient support for their applicability in our study (Phillips et al., 2014). Moreover, while deviations from assumed discrimination factors might lead to variation in predicted absolute dietary proportions, they would be unlikely to alter our conclusions on seasonal shifts in the relative importance in dietary resource among seasons. This is because we observed clear shifts in mouse tissue *δ*^13^C and *δ*^15^N values that made them more, or less, similar to the isotopic values of specific dietary resources in each season (Fig. 4). This conclusion is further supported by the observation of little to no seasonal differences observed in the isotopic values of major prey resources (Table 1, Table S2).

A key requirement of stable isotope mixing models is a degree of prior knowledge about the types of prey that may be consumed by a study species and that prey sources are isotopically distinct (Phillips et al., 2014). For example, in isolation stable isotope analysis cannot conclusively determine if mice on SEFI are consuming endemic arboreal salamanders. This mixing model approach only provides their possible dietary contribution if an *a priori* assumption is made that mice on SEFI do consume salamanders. Given the similar stable isotope values of salamanders and arthropods, the stable isotope mixing model used in these analyses had difficulty distinguishing the relative importance of these two resources to mouse diets when both were included in the analyses. This is evident by a strong negative correlation (*r* =  − 0.81) between these two group’s predicted dietary contributions and broadly overlapping 95% CI (Parnell & Inger, 2016; Table 3). Therefore, while our mixing models results indicate that it is possible that mice do consume salamanders on SEFI, it is not possible to establish this definitively. Similarly, the isotopic similarity of native *vs.* non-native plants as well as camel crickets *vs.* other arthropods on SEFI precludes the ability of stable isotope-based mixing models to identify the relative dietary contributions between these groups of mouse food resources (Table S2). Finally, while mixing models indicate a low contribution of intertidal resources to mouse diets, future studies on SEFI that include a greater number of trapping stations directly adjacent to the intertidal zone would be beneficial to confirm or reject this finding.

Introduced house mice may also act as competitors to native species in island ecosystems (Blázquez et al., 2019). For example, Russell et al. (2020), found mice had a strong impact on snipe (*Coenocorypha aucklandica meinertzhagenae*) through resource competition for invertebrates. While mice have been shown to have an indirect impact on adult ashy storm-petrel populations *via* hyper-predation by owls on SEFI (Nur et al., 2019), no studies to date have explored the potential for resource competition among mice and other native fauna on SEFI. Our study used an isotopic niche approach to compare the trophic niches of mice and salamanders on SEFI to gain insights into the possibility of competitive interactions between these two species. Specifically, we used *δ*^13^C and *δ*_15_N values in bi-variate space as a proxy of Hutchinson’s (1959) n-dimensional niche space defined by both scenopoetic (*e.g.*, basal carbon/habitat use) and bionomic (*e.g.*, trophic level/diets) niche axes, respectively (Newsome et al., 2007). Similar to Aslan & Polito (2021), we interpreted isotopic niche area as describing the range of dietary resources and habitats used by mice and salamanders, while isotopic niche overlap provided a measure of the potential for competitive overlap between mice and salamanders.

The isotopic niche of mice did not overlap with the isotopic niche of salamanders in the spring but did exhibit overlap with salamanders in the fall. This isotopic overlap between species suggests the potential for resource competition in the fall when mouse populations are at their highest (Fig. 2) and mixing model results suggest mice are consuming a relatively higher proportion of arthropod resources (Table 3). During the same time, arthropod densities and salamander abundance are low following peak densities and counts in the winter and spring (Fig. 2). Arthropods and other invertebrates are an important component of arboreal salamanders’ diets (Bury & Martin, 1973). While it is not possible to know if lower arthropod densities are limiting salamander abundances at this time, given their isotopic overlap with mice the potential for resource competition exists. Even so, differences in isotopic turn-over rates, metabolic factors, and/or sub-habitat scale spatial variation in isotopic baselines among taxa has the potential to lead to isotopic niche overlap even when species’ resource use differs (Hette-Tronquart, 2019). Therefore, future studies seeking to quantify the diets and trophic niche overlap of mice and salamanders at SEFI would benefit from integrating stable isotope analyses with other dietary proxies such as micro-analyses of gut contents and DNA metabarcoding analyses.

## Conclusions

In conclusion, our study highlights the omnivorous and opportunistic diets of house mice on SEFI. We found that mouse diets on SEFI rapidly respond to seasonal shifts in resource availability. Plants are the most important dietary resource in the spring, the importance of seabirds is highest in the summer, and arthropods become relatively more important in the fall. In addition, during the fall months when mouse numbers are highest, the isotopic niches of house mice and salamanders overlap significantly indicating the potential for competition, most likely for arthropod prey. The trophic flexibility of house mice, in combination with dramatic seasonal shifts in this invasive consumer’s overall abundance, drive the consumptive and competitive impacts of introduced house mice on this island ecosystem.

The final environmental impact statement for the South Farallon Islands Invasive House Mouse Eradication Project concluded that without action invasive house mice will likely contribute to declines in some native species on SEFI to below the level of population viability (USFWS, 2019). While much concern has been placed on the impact mice have on native storm-petrels (Nur et al., 2019), our results provide support for the assertion that house mice impact this island ecosystem by consuming a wide range of native birds, plants, and invertebrates and competing with native wildlife such as the endemic Farallon arboreal salamander. This indicates that an expected predatory and competitive release following mouse eradication will have the potential to provide wide-reaching restoration benefits. For example, unlike many non-native species, native plants on SEFI evolved without predation pressure from mammals (Coulter & Irwin, 2005; Hawk, 2015). Therefore, eliminating mouse predation on native plant seeds and shoots will likely increase germination and survival rates of foundational, native species like the maritime goldfield (USFWS, 2019). As such, we recommend that in addition to ongoing seabird studies (*e.g.*, Johns & Warzybok, 2019) post-eradication monitoring efforts focused on plant, terrestrial invertebrate, and salamander population responses are warranted to confirm these predictions. While studies such as ours are necessary to assess the current state of invaded ecosystems and inform invasive species removal, long-term monitoring of post-eradication ecosystems are crucial to assess success, maintain biosecurity, and prevent reinvasion (Courchamp, Chapuis & Pascal, 2003).

##  Supplemental Information

10.7717/peerj.13904/supp-1 Data S1 Mouse measurements, carbon (*δ*^13^C) and nitrogen (*δ*^15^N) stable isotope values, elemental concentration, and C/N ratio of mouse tissues and major prey resource group collected on Southeast Farallon Island in 2013 These data include sampling locations, age, sex, body measurements for each individual mouse. They also include the elemental concentrations and stable isotope values of mouse liver, mouse muscle, and food resources.

10.7717/peerj.13904/supp-2 Table S1 Seasonal trends in the abundance of invasive house mice (Mus musculus), precipitation, and major prey resources on Southeast Farallon Island, CA The mean ± SD house mice (Mus musculus) trapping success (%), precipitation (cm/month), arthropod density (indiv./m2), seabird (Cassin’s auklet Ptychoramphus aleuticus) carcass abundance (indiv./month), arboreal salamanders (Aneides lugubris farallonensis) abundance (indiv./month) on Southeast Farallon Island, CA during the winter (Dec., Jan., Feb.), spring (Mar., Apr., May), summer (Jun., Jul., Aug.), and fall (Sep., Oct., Nov.) seasons using data collected from December of 2000 to January of 2018. SD are not provided when less than three months of data were available in a specific year and season.

10.7717/peerj.13904/supp-3 Table S2 The carbon (*δ*^13^C) and nitrogen (*δ*^15^N) stable isotope values and C/N ratio of potential prey resources collected on Southeast Farallon Island, CA in 2013 Major taxonomic groups that share a superscript are not significantly different at the *P* < 0.05 level.

10.7717/peerj.13904/supp-4 Table S3 The stable isotope values of major prey resources used for dietary mixing model analyses of invasive house mice (Mus musculus) on Southeast Farallon Island, CA in 2013 The carbon (*δ*13C) and nitrogen (*δ*15N) stable isotope values, elemental concentration, and C/N ratio of major prey resource group averaged across all seasons on Southeast Farallon Island, CA in 2013 and used in stable isotope dietary mixing model analyses. Groups that share a superscript are not significantly different at the *P* < 0.05 level.

## Additional Information and Declarations

### Field Study Permissions

The following information was supplied relating to field study approvals (i.e., approving body and any reference numbers): Sampling was approved by the United States Fish and Wildlife Service under a cooperative agreement with Point Blue Conservation Science (no. 81640AJ008) and the California Department of Fish and Game scientific permit (no. SC–008556).

### Data Availability

The following information was supplied regarding data availability: Raw data is available in the Supplemental Files.
