# Supplementary material for: Population dynamics and resource availability drive seasonal shifts in the consumptive and competitive impacts of introduced house mice (Mus musculus) on an island ecosystem
Source: PeerJ. 2022 Sep 22;10:e13904. doi: 10.7717/peerj.13904 (PMC9509673; doi:10.7717/peerj.13904)
Supplement: Table S1 — The mean ± SD house mice (Mus musculus) trapping success (%), precipitation (cm/month), arthropod density (indiv./m2), seabird (Cassin’s auklet Ptychoramphus aleuticus) carcass abundance (indiv./month), arboreal salamanders (Aneides lugubris farallonensis) abundance (indiv./month) on Southeast Farallon Island, CA during the winter (Dec., Jan., Feb.), spring (Mar., Apr., May), summer (Jun., Jul., Aug.), and fall (Sep., Oct., Nov.) seasons using data collected from December of 2000 to January of 2018. SD are not provided when less than three months of data were available in a specific year and season. [file peerj-10-13904-s002.docx]

**Table S1**

| Year | Season | Mice | Precipitation | Arthropod | Seabird | Salamander |
| --- | --- | --- | --- | --- | --- | --- |
| 2001 | Winter |  | 7.5±8.3 |  |  |  |
| 2001 | Spring | 0.09±0.06 | 0.7±1.2 |  | 12.0±11.5 |  |
| 2001 | Summer | 0.29±0.07 | 0.2±0.4 |  | 40.7±47.7 |  |
| 2001 | Fall | 0.78±0.06 | 12.3±9.4 |  |  |  |
| 2002 | Winter | 0.31±0.33 | 4.8±0.9 |  |  |  |
| 2002 | Spring | 0.02±0.03 | 1.8±2.5 |  | 67.3±62 |  |
| 2002 | Summer | 0.12±0.13 | 0.1±0.2 |  | 68.0±43.4 |  |
| 2002 | Fall | 0.55±0.26 | 10.8±11.6 |  | 4.3±2.1 |  |
| 2003 | Winter | 0.37±0.27 | 4.7±1.2 |  | 5.0±1.0 |  |
| 2003 | Spring | 0.03±0.04 | 3.7±4.6 |  | 56.7±34.1 |  |
| 2003 | Summer | 0.24±0.21 | 0.2±0.3 |  | 95.3±47.6 |  |
| 2003 | Fall | 0.60±0.16 | 8.2±9.2 |  | 0.3±0.6 |  |
| 2004 | Winter | 0.22±0.18 | 12.8±9.5 |  | 2.3±4.0 |  |
| 2004 | Spring | 0.11 | 3.2±4.5 |  | 29.0±19.1 |  |
| 2004 | Summer |  | 0.0±0.1 |  | 53.0±36.9 |  |
| 2004 | Fall |  | 4.4±5.7 |  | 0.0±0.0 |  |
| 2005 | Winter |  | 12.1±3.1 |  | 1.0±1.7 |  |
| 2005 | Spring |  | 7.8±4.4 |  | 9.0±11.5 |  |
| 2005 | Summer |  | 0.5±0.4 |  | 6.0±5.6 |  |
| 2005 | Fall |  | 11.3±8.3 |  | 0.3±0.6 |  |
| 2006 | Winter |  | 12.9±6.5 |  | 1.3±0.6 |  |
| 2006 | Spring |  | 0.4±0.6 |  | 13.0±5.6 |  |
| 2006 | Summer |  | 0.0±0.0 |  | 8.0±3.5 |  |
| 2006 | Fall |  | 5.1±2.5 |  | 0.0±0.0 |  |
| 2007 | Winter |  | 4.5±2.9 |  | 0.3±0.6 |  |
| 2007 | Spring |  | 0.3±0.2 |  | 9.0±3.6 |  |
| 2007 | Summer |  | 1.5±1.8 |  | 32.7±29.1 |  |
| 2007 | Fall |  | 8.3±6.6 |  | 0.0±0.0 |  |
| 2008 | Winter |  | 4.0±5.3 |  | 0.7±0.6 | 74.8±16.6 |
| 2008 | Spring |  | 0.2±0.1 |  | 20.7±11.0 | 21.7±35.8 |
| 2008 | Summer |  | 0.3±0.3 |  | 43.3±14.6 | 0.0±0.0 |

**Table S1 (continued)**

| Year | Season | Mice | Precipitation | Arthropod | Seabird | Salamander |
| --- | --- | --- | --- | --- | --- | --- |
| 2008 | Fall |  | 3.4±1.3 |  | 1.3±2.3 | 19.5 |
| 2009 | Winter |  | 6.6±6.6 |  | 0.7±1.2 | 35.2±1.3 |
| 2009 | Spring |  | 1.0±1.5 |  | 15.0±10.5 | 22.2±33.4 |
| 2009 | Summer |  | 1.8±2.0 |  | 15.0±7.9 | 0.0±0.0 |
| 2009 | Fall |  | 6.3±6.7 |  | 2.7±2.5 | 17.5 |
| 2010 | Winter |  | 7.5±3.5 |  | 1.7±2.9 | 42.5±27.8 |
| 2010 | Spring |  | 2.8±2.3 |  | 11.7±2.5 | 41.9±20.9 |
| 2010 | Summer |  | 0.1±0.1 |  | 14.3±15.6 | 7.8±6.7 |
| 2010 | Fall |  | 4.9±4.1 |  | 0.0±0.0 | 13.0±5.3 |
| 2011 | Winter | 0.46±0.33 | 8.4±4.4 |  | 2.3±2.1 | 43.3±19 |
| 2011 | Spring | 0.08±0.01 | 5.9±7.1 |  | 18.0±17.3 | 19.2±21.8 |
| 2011 | Summer | 0.48±0.29 | 1.9±2.9 |  | 32.0±6.6 | 4.5±2.6 |
| 2011 | Fall | 0.74±0.36 | 3.6±3.1 |  | 2.3±2.1 | 12.8±16.6 |
| 2012 | Winter | 0.09±0.05 | 3.5±2.8 |  | 2.7±3.1 | 35.5±21.5 |
| 2012 | Spring | 0.0±0.0 | 5.1±5.7 |  | 20.7±2.5 | 36.0±27.8 |
| 2012 | Summer |  | 0.2±0.1 |  | 45.0±11.3 | 0.7±1.2 |
| 2012 | Fall |  | 6.0±6.6 |  | 5.3±6.1 | 14.0±8.5 |
| 2013 | Winter |  | 6.3±7.9 |  | 6.0±8.7 | 35.4±12.7 |
| 2013 | Spring | 0.08 | 2.2±1.4 |  | 57.3±21.4 | 27.0±23.6 |
| 2013 | Summer | 0.37 | 0.6±0.5 |  | 57.7±50.3 | 0.0±0.0 |
| 2013 | Fall | 0.86 | 1.2±1.3 |  | 0.0±0.0 | 24.0±8.5 |
| 2014 | Winter |  | 4.9±8.5 | 3.2±0.8 | 21.3±18.7 | 26±36.2 |
| 2014 | Spring |  | 3.6±3.9 | 4.7±0.6 | 41.0±26.0 | 32.5±23.0 |
| 2014 | Summer |  | 0.2±0.2 | 1.1±0.3 | 42.0±31.4 | 0.0±0.0 |
| 2014 | Fall |  | 3.5±3.5 | 1.6±0.4 | 5.0±2.6 | 3.0±4.2 |
| 2015 | Winter |  | 12.1±17.2 |  | 9.7±9.1 | 38.2±36.8 |
| 2015 | Spring |  | 0.9±1.4 |  | 27.7±16.9 | 16.3±24.5 |
| 2015 | Summer |  | 0.2±0.2 |  | 25.0±18.7 | 0.0±0.0 |
| 2015 | Fall |  | 1.4±2.0 |  | 1.0±1.7 | 5.5 |
| 2016 | Winter |  | 7.9±5.8 |  | 6.0±2.6 | 30.5±14.3 |
| 2016 | Spring |  | 5.3±6.6 |  | 14.3±8.4 | 22.7±21.5 |
| 2016 | Summer |  | 0.2±0.1 |  | 31.3±24.2 | 0.0±0.0 |
| 2016 | Fall | 0.63±0.01 | 6.1±5.7 |  | 3.3±3.5 | 1.0±1.4 |
| 2017 | Winter | 0.16±0.03 | 15.8±4.9 |  | 7.3±6.4 | 17.7±12.7 |
| 2017 | Spring | 0.06 | 5.1±4.7 |  | 26.3±4.9 | 17.2±16.4 |
| 2017 | Summer | 0.85 | 0.3±0.2 |  | 30.7±14 | 0.0±0.0 |
| 2017 | Fall | 0.89±0.01 | 1.9±2.5 |  | 5.3±5.1 | 2.0±3.5 |
| 2018 | Winter | 0.15±0.06 | 6.8±9.2 |  | 6.5±2.1 | 15.8±9.5 |
