# Supplementary material for: Population dynamics and resource availability drive seasonal shifts in the consumptive and competitive impacts of introduced house mice (Mus musculus) on an island ecosystem
Source: PeerJ. 2022 Sep 22;10:e13904. doi: 10.7717/peerj.13904 (PMC9509673; doi:10.7717/peerj.13904)
Supplement: Table S3 — The carbon (δ13C) and nitrogen (δ15N) stable isotope values, elemental concentration, and C/N ratio of major prey resource group averaged across all seasons on Southeast Farallon Island, CA in 2013 and used in stable isotope dietary mixing model analyses. Groups that share a superscript are not significantly different at the P < 0.05 level. [file peerj-10-13904-s004.docx]

**Table S3**

| Prey sources | *n* | Carbon % | Nitrogen % | C/N | δ^13^C (‰) | δ^15^N (‰) |
| --- | --- | --- | --- | --- | --- | --- |
| Plant | 46 | 38.7±3.1 | 3.4±1.1 | 12.8±4.7 | -28.2±1.5^a^ | 23.1±3.5^a^ |
| Insect | 47 | 43.7±8.5 | 14.9±2.7 | 4.9±1.0 | -22.9±2.5^b^ | 24.9±3.5^b^ |
| Intertidal | 11 | 35.9±1.6 | 9.5±0.7 | 3.7±0.2 | -11.7±1.5^c^ | 14.0±1.4^c^ |
| Seabird | 16 | 38.7±8.1 | 9.2±2.3 | 2.7±0.8 | -17.1±1.5^d^ | 13.2±2.7^c^ |
| Salamander | 32 | 30.1±4.0 | 9.1±1.3 | 3.3±0.1 | -20.7±1.4^e^ | 25.2±1.6^b^ |
